# Supplementary material for: Haplotypes of ATP-Binding Cassette CaABCC6 in Chickpea from Kazakhstan Are Associated with Salinity Tolerance and Leaf Necrosis via Oxidative Stress
Source: Biomolecules. 2024 Jul 10;14(7):823. doi: 10.3390/biom14070823 (PMC11275178; doi:10.3390/biom14070823)
Supplement: Supplementary file 1 [file biomolecules-14-00823-s001.zip › Supplementary-material-S1-S3-06-06-2024.pdf]

biomolecules

Supplementary material

Supplementary material S1. Sequences and information about primers for *CaABCC6* gene (Ca09705) used for: (1) Sequencing of fragments in open reading frame (ORF) regions and (2) RT-qPCR targeted gene and reference gene primers.

Name	Sequence 5'-3'	Amplicon size (bp)
**1. Primers for sequencing in ORF**		
Ca09705-seq-F1	ATGCTCATACAGGCTCTCACCT	1,070
Ca09705-seq-R1	GCATAAAGCACTTCCAATGGCC	
Ca09705-seq-F2	CTACAGACCAAAGTGCAGTGGA	1,171
Ca09705-seq-R2	GAGCGGAAGATGAGGAGCATAT	
**2. Primers for RT-qPCR**		
Ca09705-Fq	TATGGAGGAGCTCTTGTACCGT	109
Ca09705-Rq	CATCTGCTGAAATGGGAGTAGC	
CaELF1-Fq	TCCACCACTTGGTCGTTTTG	64
CaELF1-Rq	TTAATGACACCGACAGCAACAG	

Supplementary material S2. DArT raw-data are present in separate Excel-file.

Supplementary material S3. Full sequence of *CaABCC6* (Ca09705) genes from reference genome cv. Frontier with indicated positions of seven SNPs (Figure 5) and positions of primers (Supplementary material S1 above). The genetic polymorphism and the distribution of the *CaABCC6* alleles among haplotypes are indicated in the Table below, using the same colours for SNP identification as present in Figure 5.

>Ca2: 35,444,913-35,452,674 (CDS with 1 Kb flanking fragments) from LIS database in reverse-compliment order. 'Start-codon' is indicated by green. 'Stop-codon' is by red. Exons are by yellow. Introns and untranslated regions (UTR) have no colour. Primers for sequences and RT-qPCR are indicated by dark-grey and purple, respectively.

AATCACTAATCCCCATCAATCATTTTGAGTTTTCGTCTCTTCATTTCAGTGAAAAACCACAAACTCT
CTGTCACAGTCTCTGTCTCTTTCTAACTTCCTCTTGGTTGTTTTGTCTCTTCTTTTTTTTTTTTTTT
TTTTTTTTTTTTTTTTTTTTTTTTTTTTTTCCTATTTTTTTTTTTTTTTTTTTTTTTTTTTTTTTTT
TTCTCTTTATTATTTTCGTAGTTTCTTCCTTAGTATATTCCGTCGTCCACTATACTCAATTCTTGGCC
ACCACGTTAACCCTATTCCCAAGGAACCTCAATTTTTCCTTTGACCCTCTTTACAATTTTGACTTCAA
TACTTCATTACTCACACAACTTTTCCACCCTTTATTTATAAACCTCAAAAATTTCATCTTTCAAACTA
TCTCGTTAAACCCTTTTCTTCACTGAACCTCACCAATAAGCTTTTGCCAAAAAAAATACATTTTTGTT
TTACTATGTTGTTTGTTTTGTCTTCTATTTCACTCACGAACTTTTCTACTGATTTTCTTCTAAAACCC
ATTTTCCTTCATGGGTTATCTAGTATTCTCCATCTAGTGTTGCTTGTTGGGGTTTTGGTTTCATGTGT
TTGGAAAAAAATCACAACTTGTGTTGTGAATGAGTCTGAGAAAAAATATAGTAATACTTTGTTTAAAG
TGACAAAGTTTTGTTCTTTTGGTTTTTCTTCTTTCAATTTTGTTCTGTTTCTGTTTAATTGCTTCTAC
TGGTATACTAGTGGTTGGCCAGAAGAAAAAGTTGTTACCCTTTTTGATTTAGCTGTCAAAACAGTTGC
TTGGTGTGTTGTTTGTGTTTGTTTCCACAAAGGGTTCTTCTTTTTCTTGAGTTCATGTCAAAGAAGAA
GGTTTCCATTTTTCTTCAGAGCTTGGTGTGTTTTCTATCTCTTTGTTTCATGTTATTGCTTTGTGGTG
GACATTGTTGTTCTTTATGAATTTCATGTTGCTTTAACTGCTCAATGCATGGTTTCTGATGTGGTATC
TGTTTGTGTGAGTTTGTTCTTCTGTTATGTGGGATATTTTGTGAAAAGTAGGAGTGAAGAAGGTGATA
GAACTCTTCAAGAGCCTCTTTTGAATGGTGGTTCACATGTTGGTAATGGTGATGATAAAGTTAATGCT
TTGGATTTAAAAGAGACCAAAGGAAGTGACACTGTTACCCCTTTCTCAAATGCTGGAATTTTGAGCCT
TCTTACCTTCGCTTGGGTGGGGCCCCTTATAGCAGTTGGCAATAAGAAAACCTTGGACCTTGAGGATG
TTCCTCAGTTGGATAGTGGAGATAGTGTGTTTGGAGCTTTTCCAACTTTTAGAGACAAACTTGATGCT
GATTGTGGTGCAATCAATAGAGTAACCACACTTAAGTTGGTGAAGTCATTAATAATCTCAGGGTGGAA
AGAAATTCTTTTCACTGCTTTTCTTGCATTGATAAACACTTTTGCTTCTTATGTTGGTCCTTATCTTA
TTGATTCTTTCGTTCAATACCTTGATGGACAAAGGCTATATGAGAATCAAGGCTATGCCTTGGTTTCT
GCATTCTTCTTTGCAAAGCTTGTAGAGTGTTTTACACAAAGACATTGGTTCTTTAGGTTGCAGCAACT
CGGACTTCGAATTCGAGCACTGCTCGTGACTATGATATATAATAAAGCCTTAACACTTTCATGTCAAT
CAAGGCAGGGCCACACTTCTGGTGAAATAATCAATTTCATGACTGTTGATGCTGAAAGAGTTGGTGTT
TTCAGTTGGTACATGCATGATTTATGGTTAGTAGTTTTGCAAGTTACATTGGCCTTGTTGATTTTGTA
TAAAAACCTTGGTGTTGCTTCAGTTGCTGCTTTTGCTGCAACTATCATTGTTATGTTGGCAAATGTTC
CATTAGGATCATTGCAAGAGAAGTTTCAAAGTAAGTTGATGGAGTCAAAGGATACCAGAATGAAAACA
ACATCTGAAATTTTAAGGAACATGAGGATCCTCAAACTACAAGGATGGGAAATGAAGTTTCTATCTAA
AATAACTGAGCTCAGGAACAATGAGCAAAACTGGTTGAAGAAATTTCTTTATACTTCAGCCATGACTA
CATTTGTCTTTTGGGGTGCACCTACATTTGTTTCTGTGGCTACTTTTGGCACTTGCATGCTTATAGGG
ATTCCTCTTGAATCAGGGAAGATTCTGTCAGCACTTGCAACATTCAGGATTCTTCAAGAGCCTATTTA
TAATCTTCCTGATGTTATTTCAATGATAGCACAAACTAAAGTTTCTCTTGATAGGATCGCATCGTACC
TTCGTCTCAATGACTTGCAGTCCGATGTTGTAGAGAATCTTCCTCCCGGTAGTTCCGATACAGCAATC
GAAGTGGTCGATGGAAACTTTTCTTGGGATTTATCTTCTACTAATCCAACTTTGCAGAACATAAATGT
AAGAGTTTCTCATGGCATGAAGGTTGCTGTTTGTGGTACTGTTGGATCAGGGAAATCTACTCTACTTT
CTTGTGTATTAGGAGAAGTACCAAAGATATCAGGTATCCTTAAGGTTTGTGGAACAAAGGCTTATGTT
GCTCAATCACCATGGATTCAAAGTGGAAAGATAGAGGATAATATATTGTTTGGTAAGGACATGGATAG

GGAAAGGTATGAGAAGGTTCTTGAAGCATGTTCCTTAAAGAAGGATCTAGAGATTTTGTCATTTGGTG
ATCAAACAGTTATAGGAGAGCGTGGAATAAATTTGAGTGGTGGACAGAAACAAAGAATACAAATTGCA
CGCGCTCTTTACCAAGATGCTGATATGTATCTATTTGATGATCCTTTTAGTGCTGTTGATGCTCATAC
AGGCTCTCACCTTTTCAAGGTAAATAACACATTTTCATTCTTTTGTTTATTATGTACAATATAAACTA
GGCTATTTATTTATTTTTGCATGCTGCAAAATCAATGTTGTCCAATCGTGGCTATAGCAACACTATAG
CACTACAACATAAAAGAATTTGAATACATCTTTATTGTTTTGCTATAGTGGCGCTATAGCATTGCAGC
GTAGTGGAATTTGAACAAACTGCTATCTACTTCAATCTATGATTGACAACATTGTACAAAATACAAAT
AATTTTCAAACTCTTTTGAAATGTAGCTATTCCAACTGCATTACACTTGTATAATTATTATTTTAGTA
TAAGTGAAATGGTCTGTTTTAATTATGTGACAGGAATGCTTGCTGGGTTATTTAAGTTCAAAAACAGT
TGTTTATATTACTCATCAAGTGGAGTTCTTACCTACTGCTGACCTTATATTGGTGAGTATTTATTTAT
CATAATTCTATTGTCATATCATTTATTTAAAAGGAGGAATTTTATTATAACCTTTTTATTTGACAATT
GTTGTGTAGGTCATGAAAGATGGAAAAATTACTCAAAGTGGAAAGTATGCCGATCTGCTTAACATTGG
GACTGATTTTATGGAACTTGTTGGTGCACATAGAGAAGCTTTGTCTACACTTGAAACATTGGATGGAG
GAAAAGAATCTAATGAAATAAATACATTGGAACAAGATGTAAGCATCTCTGTCAGTGTCGCTCATGAT
GTCAAAGAAAAGGAGACAATCAAAGATGAGCAAAATGATAAAGGTGAACCGAAAGGCCAACTTGTTCA
AGAAGAAGAAAGGGAGAAAGGTAAAGTTGGTTTTTCAGTGTATTGGAAGTATATCACAACTGCATATG
GAGGAGCTCTTGTACCGTTCATATTACTGGCTCAGATTCTTTTTCAATTTCTTCAAATTGGAAGCAAC
TATTGGATGGCTTGGGCTACTCCCATTTCAGCAGATGTGGAAGCACCTGTTGAAGGCACAACTCTTAT
AGAAGTCTATGTTGCTTTGGCCATTGGAAGTGCTTTATGCATTCTTGTTAGAGCATTGTTACTTGTTA
CAGCTGGTTATAAGACAGCTACTATACTCTTCAATAAAATGCACTTGTCTATTTTTCGTGCTCCGATG
TCATTTTTTGATTCTACTCCAAGCGGTCGAATCCTTAACCGAGTATGTCATATCGATACCCTTGATTA
TTTCCATTTGTTTCCCATTTGATTTCCAGAGATATGATTTTTGCATCTTAAATTGCTCTAGACGTCCC
TTACATTTTGTTAGTTGTTTTAAAAAACTATGCAATACTTTGTCATCGCTTGTTTTTTTATCTCTTTC
TTCTGCTTCGCTATCTTGCAGGCTTCTACAGACCAAAGTGCAGTGGATACAGACATTCCTTATCAAAT
TGGTTCATTTGCCTTCTCCTTGATCCAACTTTTCGGAATCATAGTAGTGATGTCCCAGGTTGCATGGC
AAGTGTTCATTGTTTTTATACCCGTGATAGCCATCAGCATTTGGTATCAGGTATTATTTTGAACCATC
AATCATGTCTAGGCTTTTGATAACAAGTTGGCCCATTTTTTGTGCAAATTTGATTCCAGTGTATAATA
TTATATGGCGATGCTTGACAACTTTGGAACTTGTAATCTGCAGCGATTTTACTTACCATCAGCACGTG
AACTATCACGTTTGGTTGGAGTGTGCAAAGCTCCAATCATTCAACACTTTGCAGAAACAATTTCTGGT
ACTACAACCATTAGAAGCTTTGGTCAACAGTCGAGATTTCATGAAACGAATATGAAACTGACCGATGG
GTATTCTCGTCCAAAGTTCAATATTGCTGCTGCTATGGAATGGTTATGCATCCGCTTAGATATGCTGT
CTTCCATCACATTTGCCTTTTCCTTGATATTCTTGATATCTATTCCACAGGGAATCATAAATCCAGGT
GCATAATTTGAATCTGTGGTATCATATATGAATCAAAATTTACTCTTCTTATTATGAAATAAGGAGTT
GAGCATAATTGTTTCCATTTTGTGTCATTTTTTCTGATTTTTGAAGGCATTGCTGGTCTAGCTGTTAC
CTATGGTCTAAATTTAAACATGATACAAGCTTGGGTGATATGGAATCTTTGCAACTTGGAGAACAAAA
TTATATCGGTAGAAAGGATGCTTCAGTATACAAACATTCCTAGTGAGCCTCCTCTAGTTTTAGAAGAA
GAAAATAGACCAGATCCTTCTTGGCCATCATACGGCGAGGTTGATGTACGGAACTTGCAGGTATAGGA
CTTTTTTCTTCCTGTCGTTGCCTTTTGTTTATGTCTTTGGTTTTGTTTTCTTTCATTTTGTGGTCAAT
TGATTTATGAAACTCATGTTAATGTTTTCTTTTCGTAAATGGTTAGAAAAGTTGTGATTTGGATATAA
CATACTTGTTAGCATAGTTTCTTTACTAGATGTTTGTGTTCTGTTTCACCATTTGTAATGACTTGTT
TTCTGTGATGAAGGTTCGATATGCTCCTCATCTTCCGCTCGTGTTGCGTGGCCTGACATGCACATTTC
GTGGAGGATTGAGAACTGGCATTGTTGGTAGAACAGGTAGCGGCAAATCAACTCTTATACAAACACTA
TTTCGACTTGTTGAACCTACTGCCGGGGAAGTTATCATAGACGGCATCAACATATCTACAATTGGGTT
GCATGATTTGAGGTCTAGACTAAGCATTATTCCCCAGGATCCGACAATGTTTGAGGGGACTGTGAGAA
GTAATCTCGATCCTCTGGAAGAGTACACCGATGAACAAATATGGGAGGTGGGTTGGTGTTTTATATTT
GTTGGGAAAAAATGAAATGACAATGACACTTAGTGTTCTGTTCAAAATGTTCGACATGTAATTGTTCT
TCAAATACTGATTTACATTCTCTAAATTTAAAATAGGCCTTGGATAAGTGTCAACTGGGAGATGAAGT
TAGAAAGAAAGAAGGAAAGCTCGATTCTGCAGGTTTGTCTTCTAATCAATGTTTTCAATCATGGATCA
TGAAAAATAGCAGTTTGTTTAAATTTTGTTACACTACAGTGCCACTATAGTTGCGCTAAATAGTGTAT
TGCAGAATCATAGTTATTTGTATAAATTTGGCTACACTATAGTGGCTATAGCTATAGCCACTATTTGA
CAACAATGCTTCTAATTTTGTTGAACACAAATTAATAGTGCTTGTACTCTTGAACTTATTTCTAATGT
GGAAGTTGATGCTTTTATAGTTAGTGAGAATGGTGATAATTGGAGCATGGGTCAGAGGCAGTTGGTTT
GTCTTGGTAGGGTTCTTCTTAAGAAGAGCAAGATTTTGGTGCTTGATGAAGCCACTGCATCAGTTGAC

ACAGCTACAGATAATTTAATTCAGCAAACTCTTAAGCAGCATTTCTCCGACTCTACCGTCATTACCAT
CGCACATCGAATAACTTCTGTTCTTGACAGTGATATGGTTCTACTTCTTAGTCAAGGTCAGTAACAAA
AACTATTATTCGTAGTTTAAAATTTGATATCTTTTGTTCTTTTGGTGTTTCGTTTTTGAATTTAGATT
GCCTGCAATCAGCAATACCATAACCAGTACATTGGTGACTGTTGAATCGAAATTAAACAGCCCAAATT
TTGAACTCAGTATTTTTAGTATATTATTTTATAAAAATTAAAATATGCATTGAAATCTGGACCGTCCG
ATCTCAATTTCGTTTTCTAATCTTGTTGTGTATTGGTGTAGGGCGTATTGAGGAGTATGACTCTCCAA
CCACATTGCTAGAGGATAAGTCATCATCTTTTGCTAAGCTTGTTGCAGAGTATACCATGAGGTCCAAC
TCCAGTTTTGAGAAATCTGTTCATCAATGATTGAAGTCCACTGAAATATATTTTTTTTGATGATGATG
AAAATCTGGGGGTGGGGTGGGGATACCAGTATCTTATTAATGGTTTTTTGATAGATAAATGTAATATA
TAATAATAAGGTTTTGGATTTCCAGTTTCACCTTTTCTTCCCAAGGCTGAAGTAAATTATTAGAGGGA
AGAATTATAAAATGAAAGAAAATCTAGTTACTTTCAATGCATTAATTTAATGTGAATAGTAACTACAT
TATTATTTTTACAATGCATCTATGCAAGTGCAATTATATTCATATTAAAAGCAAAATTGACAGACATA
CTAGGACGTTTTTTTTATTTTTTTATCTTAATCTTTTTGACCAAAGATTGAACACTAACTTGCATATA
AAAGTAGTATATCAATCTCATGACAACTTAAGCCTTTCTAGTTGTATGAATTTTAGAAGTTGAAATCA
TTTTTGAATATTCTTTACAAGTTGACAATCAAATAAACAAATATAACTAATCATTGTATAAATACAAC
AAAATCAAGCACAAAATTATACACCACCAATCTACATTGCTTGTCAAATTTAATTATCCCAAATCTTT
AAGATTTAATTAAATTGACAAATACTGATATTATTAAGTTGAATATATCAACTTAAGCCTTTTTAGTT
GTATGAATTTTAGAGCTACTTGTTCCTCCATTTACTATTAAACAAATTAATTCAATTTCACCGGAACC
CGCAAAACCAAAATCCACTCCAATCAATGCACAAGAACTGACTCACCAAAAACCTCTTGACCGATATG
TTAACACCAGAGTGCTATTCCACCATGACTCTTGCATTATGCTACATGAAACAAAGAATCTACACACA
AATTGTGATTCCCATAAAAAATATCATTCAAAGGAAACTCTGATAGTCAGGGGTTCTAAGAGGAATAA
AACCAAACCTAATTTGTACCAAAACCAATCCTACCATATACATGGTTTGCTGAACAATCTGAATTATT
CCTATCATAT

SNP No.	SNP allele-1, haplotypes	SNP allele-2, haplotypes	SNP position in genomic DNA from 'Start codon' (bp)	SNP position in translated CDS from 'Start codon' (bp)	SNP position in genomic DNA as it present in Table S3, Basu et al., 2019 [77]
1	**[G]** = A, B, C and D1	**[A]** = D2	2,542	2,095	Absent
2	**[G]** = A, B, D1 and D2	**[T]** = C	3,533	2,774	SNP_Ca2:35448142 [C/A]
3	**[A]** = A, D1 and D2	**[T]** = B and C	3,549	2,790	SNP_Ca2:35448126 [T/A]
4	**[G]** = A, D1 and D2	**[C]** = B and C	3,657	2,898	SNP_Ca2:35448018 [C/G]
5	**[T]** = A, D1 and D2	**[C]** = B and C	3,662	2,904	SNP_Ca2:35448013 [A/G]
6	**[T]** = A and B	**[C]** = C, D1 and D2	3,699	2,940	SNP_Ca2:35447976 [A/G]
7	**[T]** = A, D1 and D2	**[G]** = B and C	3,709	2,950	SNP_Ca2:35447966 [A/C]
